# Age and Clinical Activity Score (CAS): Key Predictive Factors for Non-shrinking Extraocular Muscles in Graves’ Ophthalmopathy After Retrobulbar Injection of Glucocorticoids (GCs)

**DOI:** 10.7759/cureus.91353

**Published:** 2025-08-31

**Authors:** Yuying Xiang, Rui Li, Guang Zhao, Fagang Jiang, Junjie Yang

**Affiliations:** 1 Department of Ophthalmology, Wuhan Union Hospital, Wuhan, CHN; 2 Tongji Medical College, Huazhong University of Science and Technology, Wuhan, CHN

**Keywords:** extraocular muscle, graves’ ophthalmopathy, predictive factors, retrobulbar injection, treatment response

## Abstract

Purpose: To identify predictive factors for non-shrinking extraocular muscles (EOMs) after retrobulbar injection of glucocorticoids (GCs) in patients with Graves’ ophthalmopathy (GO).

Methods: This retrospective cohort study included 60 GO patients (102 eyes) who received retrobulbar GC injections. We used univariate analysis, Least Absolute Shrinkage and Selection Operator (LASSO) regression, and multivariate logistic regression to identify factors associated with non-shrinking EOMs. The primary outcome was the change in EOM volume assessed by orbital computed tomography (CT) scans before and after treatment.

Results: After retrobulbar injection of GCs, 36 eyes (35.3%) showed a reduction in EOM volume, while 66 eyes (64.7%) did not. Multivariate analysis revealed that age (OR = 1.09, 95% CI: 1.01-1.18, p = 0.03) and Clinical Activity Score (CAS)≥3 (OR = 0.11, 95% CI: 0.02-0.59, p = 0.008) were independent predictors of non-shrinking EOMs. Higher age increased the odds of non-shrinking EOMs by 9% per year, while higher disease activity (CAS≥3) was associated with a better treatment response. Other factors, like proptosis and axial orbit depth, showed trends but did not reach statistical significance.

Conclusion: Age and disease activity (CAS≥3) are significant predictors of non-shrinking EOMs after retrobulbar GCs injection in GO patients. These findings can help clinicians better assess treatment outcomes and optimize therapeutic strategies for GO patients. Future research should focus on expanding the sample size and exploring underlying biological mechanisms to further validate these findings and improve treatment precision.

## Introduction

Graves’ ophthalmopathy (GO) is an autoimmune orbital disease and the most common extrathyroidal manifestation of Graves’ disease [1]. It is characterized by edema, hyperplasia of adipose tissue, and accumulation of glycosaminoglycans and collagen, which together lead to enlargement of the extraocular muscles (EOMs) and orbital fat [2,3].

Glucocorticoids (GCs) are the main treatment for GO. Due to their fewer systemic side effects, retrobulbar GC injection is gaining attention. A prospective study showed that injecting triamcinolone (TA) into the lower outer quadrant of the orbit effectively relieved diplopia in recently-onset GO patients and reduced EOMs volume, with no systemic or ocular adverse reactions observed [4].

In addition, a retrospective study involving 386 cases showed that the combined use of retrobulbar TA and dexamethasone is an effective treatment for mild to moderate GO, with varying degrees of relief for oculomotor disorders, diplopia, and strabismus, and minimal adverse reactions. The efficacy of this therapy is influenced by factors such as gender, smoking history, and disease severity [5].

Retrobulbar injection of TA for GO is relatively safe [6], but the factors that affect the treatment efficacy have not been fully identified. Identifying these factors is crucial for optimizing treatment strategies and improving patient outcomes. Previous studies have emphasized the importance of various demographic, clinical, and radiological parameters in predicting treatment response. However, the heterogeneity of GO and the complex interplay among these factors necessitate further research to clarify their individual and combined effects on treatment outcomes.

We aim to identify potential factors associated with non-shrinking of EOMs after retrobulbar GCs injection via exploratory analysis. This method can provide hypotheses for future research and reveal clinical characteristics that may affect treatment outcomes in GO patients. By focusing on exploratory analysis, we hope to better understand the individual contributions of each factor. Our goal is to offer guiding insights for future research and clinical decision-making for GO patients.

## Materials and methods

Materials

This study is a retrospective cohort study. We collected clinical data from 60 patients (102 eyes) who received GC injections at the ophthalmology clinic of Union Hospital, Tongji Medical College, Huazhong University of Science and Technology in China. Data collection took place from January 1, 2015, to January 1, 2025.

In this study, the retrobulbar corticosteroid injection regimen included 20 mg TA and 2.5 mg dexamethasone. The TA dose was selected based on previous studies [4,5], while the dexamethasone dose was determined by clinical experience. All injections were performed by the same physician using disposable syringes with needles sized 22 - 27 and a length of 3 cm.

The procedure involved inserting the needle vertically into the outer-lower quadrant of the orbit. When a depth of approximately 1.5 cm was reached, the needle was redirected medially and superiorly, and then advanced another 1.5 cm to the top of the orbit. Intraocular pressure (IOP) measurements were recorded both before the injection and on the day following the injection. A baseline orbital computed tomography (CT) scan was performed before the first injection, and subsequent injections were administered at monthly intervals, for a total of three injections. After the completion of the three injections, a follow-up orbital CT scan was conducted to assess the treatment effect.

The inclusion criteria for this study were as follows: (a) age between 18 and 80 years; (b) patients diagnosed with GO, with changes in diplopia symptoms within the past 3 months, and CT scan showing enlargement of one or more rectus muscles; (c) having received retrobulbar GCs injection during hospitalization in the ophthalmology department of our hospital; (d) no other systemic diseases except for thyroid-related diseases; (e) having undergone an orbital CT scan within 1 week before the first retrobulbar GCs injection and again within 2-3 months after the last injection; (f) Receiving low-dose (0.5 mg/kg) oral methylprednisolone tablets for maintenance treatment after injection, with a dosage reduction of 5 mg per week, for a total duration of 4 weeks; (g) IOP of the treated eye ranging from 10 to 21 mmHg before injection.

Conversely, the exclusion criteria were as follows: (a) history of ocular trauma or surgery; (b) use of GCs or radiotherapy within the past 6 months; (c) pregnancy in female patients; (d) lack of relevant data from general examinations or CT scans; (e) IOP exceeding 21 mmHg either before or after any injection.

Data collection

The health records and CT images of the enrolled patients were obtained through the electronic medical record system of Union Hospital, Tongji Medical College, Huazhong University of Science and Technology. The CT data were acquired via the Picture Archiving and Communication System (PACS), with window level/window width set at 60/300 Hounsfield units (HU) to optimize the visualization of the EOMs. Each investigator measured the diameter of each EOM three times and took the average to ensure accuracy and reduce measurement variability. Data collection and management were conducted using the Electronic Data Capture and Management System (EDC, study.empoweredc.com, Shanghai, China).

In axial CT scans, proptosis was defined as the vertical distance from the outermost point of the eyeball to the zygomatic arch line. The axial CT scans measured the horizontal diameters of the belly of the medial rectus muscle (MRM) and the lateral rectus muscle (LRM). In sagittal CT scans, the vertical diameters of the widest parts of the superior rectus muscle-levator complex (SRLC) and inferior rectus muscle (IRM) were measured, with the superior rectus and levator muscles considered as one muscle group. Additionally, orbital depth in axial CT scans was defined as the distance between the orbital apex and the zygomatic arch line.

Two observers independently assessed changes in the EOMs (enlargement, reduction, or no significant change) by comparing pre- and post-injection CT images of GO patients. In case of disagreement, a third expert was consulted to reach a consensus.

Statistical analysis

Quantitative data are presented as mean ± standard deviation (SD) and median with interquartile range (IQR). Qualitative data are summarized using frequencies and percentages (%). The normality of all measured data was assessed using the Kolmogorov-Smirnov normality test. When the data were normally distributed, the significant differences between the two groups were evaluated using the t-test. Conversely, if the data did not conform to a normal distribution, the Wilcoxon rank-sum test was employed. For qualitative data, the chi-square test was used to assess the significance of differences between groups.

Logistic regression analysis was conducted to evaluate the impact of specific factors on patient prognosis. Variables included in the multivariate regression analysis were selected based on clinical relevance and the results of univariate logistic regression analysis. Subsequently, multivariate logistic regression analysis was performed to assess the role of each factor in the regression equation.

LASSO regression was employed to identify the most relevant predictors of non-shrinking EOMs after retrobulbar injection of GCs. This method is particularly useful for handling high-dimensional data and performing variable selection simultaneously. It works by imposing a penalty on the absolute size of the regression coefficients, which can shrink some coefficients to exactly zero, thereby eliminating the corresponding predictors from the model.

The statistical analysis was conducted using R software version 4.0.3 (https://www.R-project.org) and EasyR software (https://www.easyr.cc Solutions, Inc., Shanghai). All analyses were performed using two-sided tests, with a significance level set at p<0.05 to determine statistical significance.

## Results

Patients’ characteristics

The study enrolled 60 GO patients, involving a total of 102 eyes. The gender distribution was 31 males (51.7%) and 29 females (48.3%), with an average age of 49.28 ± 9.40 years. After retrobulbar injection of GCs, 36 eyes (35.3%) exhibited a reduction in EOM volume, while 66 eyes (64.7%) showed no change or an increase in EOM volume. A summary of the baseline characteristics of the patients is presented in Table 1.

**Table 1:** Patient characteristics of the shrinkage group vs. the non-shrinkage group Variables marked with “#” indicate that the p - value was calculated using the chi - square test (χ² test); variables marked with “†” indicate that the p - value was calculated using the t - test (t - test); variables without any mark indicate that the p - value was calculated using the Wilcoxon rank - sum test (Wilcoxon rank - sum test). Significance was denoted as *P < 0.05. BMI: body mass index; FT4: serum free thyroxine; FT3: triiodothyronine; TSH: thyroid-stimulating hormone; Tg: thyroglobulin; anti-TPO: anti-thyroid peroxidase antibody; TgAb: anti-thyroglobulin antibody; TRAb: thyroid hormone receptor antibody; CAS: Clinical Activity Score; IRM: intraretinal myelination

Variables	Shrinkage Group	Non-shrinkage Group	Statistics	p
Numbers	36 (35.29%)	66 (64.71%)	-	-
Age (years)	46.11 ± 11.39	51.77 ± 8.53	828.5	0.01*
Sex= Female^#^	23 (63.89%)	28 (42.42%)	3.48	0.06
BMI	23.22 ± 2.64	23.52 ± 3.48	635	0.49
Underwent I131 treatment: Yes^#^	7 (19.44%)	15 (22.73%)	0.02	0.89
Smoking: Yes^#^	14 (38.89%)	5 (7.58%)	13.07	<0.01*
FT4 (pmol/L)	12.67 ± 4.36	13.42 ± 3.28	734.5	0.10*
FT3 (pmol/L)	4.84 ± 3.31	4.26 ± 1.16	847	0.50
TSH (μIU/L)	2.45 ± 3.10	1.65 ± 1.99	1016	0.46
Tg (μIU/L)	41.37 ± 46.84	45.93 ± 106.50	762.5	0.12
anti-TPO (IU/L)	229.54 ± 372.61	173.18 ± 325.25	873.5	0.76
TgAb (IU/L)	105.26 ± 231.44	72.75 ± 199.29	634	0.29
TRAb (IU/L)	8.17 ± 12.55	8.11 ± 12.27	683	0.71
Axial orbital depth (mm)^†^	40.75 ± 2.24	40.17 ± 1.69	1.48	0.14
Pre-injection				
Ocular prominence (mm)^†^	20.64 ± 3.77	19.79 ± 3.11	1.84	0.23
SLRC diameter (mm)	5.94 ± 2.18	5.69 ± 1.83	1161	0.87
IRM diameter (mm)	7.01 ± 1.88	6.31 ± 1.84	1408	0.05
MRM diameter (mm)	6.83 ± 2.52	5.74 ± 2.16	1514.5	0.02*
LRM diameter (mm)	4.62 ± 2.02	4.16 ± 1.49	1320	0.36
CAS score	3.11 ± 1.95	2.83 ± 2.00	1255.5	0.54
Post-injection				
Ocular prominence (mm)^†^	19.99 ± 3.30	18.81 ± 2.95	1.22	0.07
SLRC diameter (mm)	5.35 ± 2.03	5.50 ± 1.50	977	0.25
IRM diameter (mm)	5.56 ± 1.51	6.19 ± 1.72	903.5	0.07
MRM diameter (mm)	5.51 ± 2.18	5.90 ± 2.33	1071	0.41
LRM diameter (mm)	4.04 ± 1.70	4.31 ± 1.54	1035.5	0.29
CAS score	2.53 ± 1.98	2.79 ± 1.87	1016.5	0.46

Significant differences were observed in the baseline characteristics between the two groups. Compared with the non-shrinking group, patients in the shrinking group were significantly younger (46.11 ± 11.39 years vs. 51.77 ± 8.53 years, P = 0.01). Moreover, the proportion of patients with a smoking history was significantly higher in the shrinking group (38.89% vs. 7.58%, P < 0.001). In terms of laboratory tests, no significant differences were found between the two groups in levels of FT4, FT3, TSH, Tg, anti-TPO, TgAb, and TRAb. Regarding orbital parameters, the MRM diameter was significantly larger in the shrinking group (6.83 ± 2.52 mm vs. 5.74 ± 2.16 mm, P = 0.02), while other orbital parameters, such as proptosis, axial orbital depth, SRLC diameter, IRM diameter, and LRM diameter, showed no significant differences between the two groups. There were also no significant differences in CAS scores between the groups.

Exploratory analysis of factors associated with non-shrinking EOM after retrobulbar injection of GCs

We conducted a univariate logistic regression analysis to explore potential factors associated with the absence of shrinking in EOMs following retrobulbar injection of GCs in patients with GO. The results are summarized in Table 2.

**Table 2:** Univariate analysis of EOM non-shrinkage after retrobulbar injection of GCs Outcome:0=EOMs shrinking 1=EOMs non-shrinking.*P＜0.05 EOM: extraocular muscles; GCs: glucocorticoids

Factors	Odds Ratio	95%CI	p
Age(years)	1.06	1.02-1.11	0.01*
Sex=Female	2.40	1.03-5.60	0.04*
BMI	1.03	0.89-1.19	0.69
Underwent I^131^ treatment: Yes	1.21	0.44-3.37	0.70
Smoking: Yes	0.13	0.04-0.40	< 0.01*
FT4 (pmol/L)	1.06	0.93-1.21	0.36
FT3 (pmol/L)	0.89	0.72-1.09	0.26
TSH (μIU/L)	0.88	0.74-1.05	0.15
Tg (ng/L)	1.00	0.99-1.01	0.84
Anti-TPO (IU/L)	1.00	1.00-1.00	0.47
TgAb (IU/L)	1.00	1.00-1.00	0.51
TRAb (IU/L)	1.00	0.96-1.04	0.98
Ocular prominence (mm)	0.89	0.77-1.01	0.07
Axial orbital depth (mm)	0.85	0.68-1.06	0.14
SRLC diameter (mm)	0.94	0.76-1.16	0.5
IRM diameter (mm)	0.82	0.65-1.03	0.08
MRM diameter (mm)	0.82	0.68-0.98	0.03*
LRM diameter (mm)	0.85	0.67-1.08	0.19
CAS score	0.93	0.75-1.15	0.49
CAS≥3: Yes	0.42	0.18-0.98	0.04*

The analysis revealed that the following factors were significantly associated with non-shrinking of EOMs: Age: For every 1-year increase in age, the risk of non-shrinking of EOMs increased by 6% (OR = 1.06, 95% CI: 1.02-1.11, P = 0.008). This suggests that age may be an important factor influencing the reduction of EOMs; Sex: Compared with male patients, female patients had a higher risk of non-shrinking of EOMs (OR = 2.40, 95% CI: 1.03-5.60, P = 0.040). This indicates that sex may play a role in the reduction of EOMs; Smoking history: A history of smoking was significantly associated with a reduced risk of non-shrinking of EOMs (OR = 0.13, 95% CI: 0.04-0.40, P < 0.001). This may suggest that smoking has a protective effect on changes in EOM volume, although further research is needed to confirm its mechanism; MRM diameter: Patients with a smaller MRM diameter had a higher risk of non-shrinking of EOMs (OR = 0.82, 95% CI: 0.69-0.98, P = 0.028). This suggests that MRM diameter may be an important anatomical factor influencing the reduction of EOMs; CAS≥3 group: Patients with a CAS score of ≥3 had a lower risk of non-shrinking of EOMs (OR = 0.42, 95% CI: 0.18-0.98, P = 0.042). This may indicate that higher disease activity is associated with a better treatment response.

Other factors, including levels of FT4, FT3, TSH, Tg, anti-TPO, TgAb, and TRAb, as well as proptosis, axial orbital depth, SRLC diameter, IRM diameter, and LRM diameter, did not show a significant association with non-shrinking of EOMs (P > 0.05).

Least absolute shrinkage and selection operator (LASSO) regression analysis results

To identify the most relevant preoperative variables associated with the outcome, we conducted a LASSO regression analysis. The lambda.min column indicates the coefficient values at the lambda value that minimizes the cross-validation error, while the lambda.1.se column indicates the coefficient values at the largest lambda value within one standard error of the minimum error. Variables with non-zero coefficients at lambda.min were considered significant predictors of the outcome. The model selected the following variables: Age, Smoking, TSH, TgAb, CAS≥3 group, Ocular prominence, Axial orbital depth, and MRM diameter. The results of the LASSO regression analysis are presented in Table 3. 

**Table 3:** LASSO Regression Analysis Results lambda.min: Lambda with Minimum Cross-Validation Error; lambda.1.se: Lambda with One Standard Error of the Minimum Cross-Validation Error. LASSO: Least Absolute Shrinkage and Selection Operator

Variable	lambda.min	lambda.1.se
Sex=Female	0.00	0.00
Age (years)	0.06	0.04
Underwent I^131^ treatment	0.00	0.00
Smoking: Yes	0.00	0.00
FT4 (pmol/L)	0.00	0.00
FT3 (pmol/L)	-0.03	0.00
TSH (μIU/L)	-0.11	-0.07
anti-TPO (IU/L)	0.00	0.00
Tg (ng/L)	0.00	0.00
TgAb (IU/L)	0.00	0.00
TRAb (IU/L)	0.00	0.00
CAS score	0.00	0.00
CAS≥3: Yes	-1.32	-0.92
Ocular prominence (mm)	-0.21	-0.17
Axial orbital depth (mm)	0.00	0.00
SRLC diameter (mm)	-0.16	-0.09
IRM diameter (mm)	0.00	0.00
MRM diameter (mm)	0.00	0.00
LRM diameter (mm)	-0.03	-0.02

**Figure 1:**
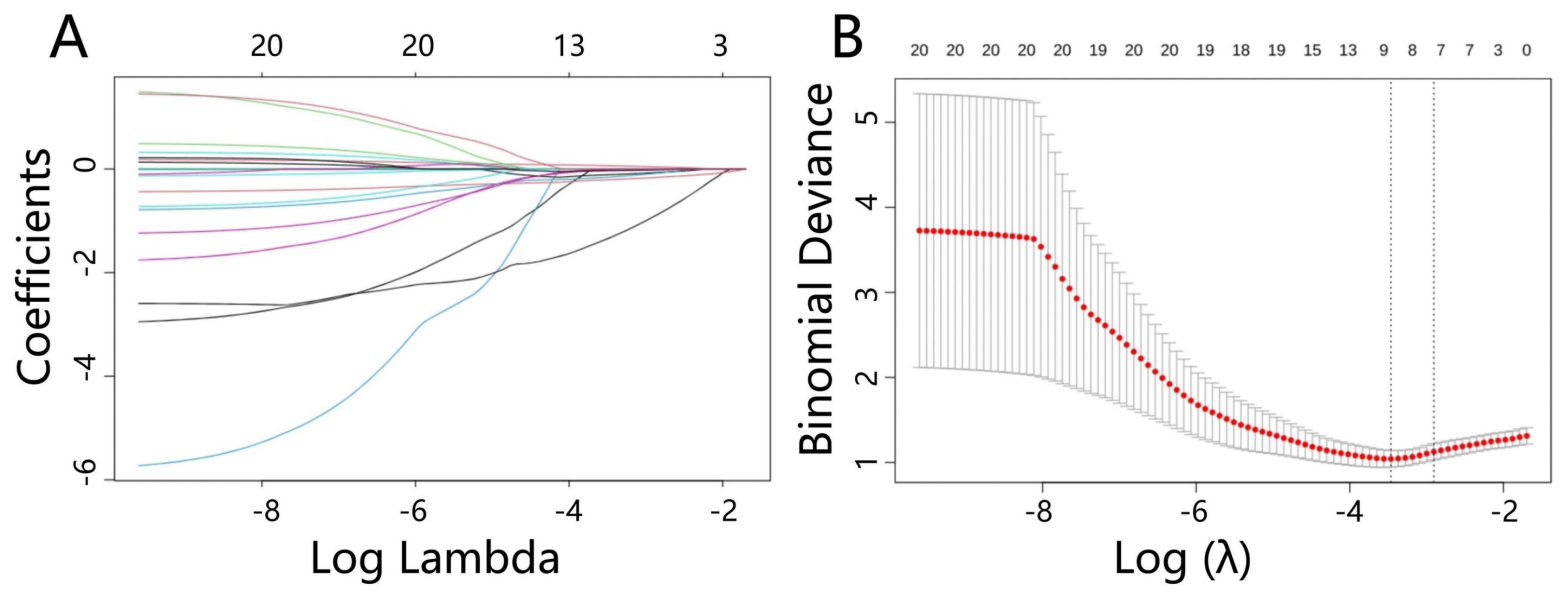
LASSO Regression Coefficient Path A: The coefficient path of the LASSO regression analysis. B: The variables selected by the LASSO model at different levels of regularization. LASSO: Least Absolute Shrinkage and Selection Operator

Figure 1 presents the results of the LASSO regression analysis, which was conducted to identify the most relevant preoperative variables associated with the outcome. Panel A displays the coefficient path, showing how the standardized coefficients of the predictors change with increasing regularization (log(lambda)). The vertical dashed line indicates the log(lambda.min) value, where the model achieves the minimum cross-validation error. Panel B highlights the variables selected by the LASSO model at this optimal lambda value. These variables, which have non-zero coefficients at log(lambda.min), are considered significant predictors of the outcome. 

Multivariable logistic regression analysis

To further investigate the independent associations between the identified factors and the absence of shrinking in EOMs following retrobulbar injection of GCs, we conducted a multivariable logistic regression analysis (Table 4). We further evaluated the independent effects of age, sex, smoking history, TSH, TgAb, TRAb, CAS≥3, proptosis, axial orbit depth, and MRM diameter on the non-shrinking of EOMs after retrobulbar injection of GCs. These factors were included in the multivariate model for adjustment because they showed significance or clinical relevance in the univariate analysis, or were selected in the LASSO regression. TRAb, which is closely related to the occurrence of GO, was also included in the multivariate model.

**Table 4:** Multivariable Logistic Regression Analysis of Factors Associated with Non-shrinking EOM after Retrobulbar Injection of GCs Outcome:0=EOMs shrinking 1=EOMs non-shrinking. CAS: Clinical Activity Score; EOM: extraocular muscles *P＜0.05

Factors	Odds Ratio	95%CI	p-value
Age (years)	1.09	1.01-1.18	0.03*
Axial orbital depth (mm)	0.74	0.52-1.04	0.08
Ocular prominence (mm)	0.77	0.60-1.00	0.05
CAS≥3: Yes	0.11	0.02-0.59	0.008*
TSH (μIU/L)	0.78	0.60-1.02	0.06

The analysis revealed that age (OR = 1.09, 95% CI: 1.01-1.18, p = 0.03) was significantly associated with the risk of non-shrinking of EOMs, indicating that for every 1-year increase in age, the odds of EOMs not shrinking increased by 9%. Clinical activity score (CAS≥3, OR = 0.11, 95% CI: 0.02-0.59, p = 0.008) was also significantly associated with a lower risk of non-shrinking of EOMs, suggesting that higher disease activity may be related to a better treatment response. Axial orbit depth (OR = 0.74, 95% CI: 0.52-1.04, p = 0.08) and TSH level (OR = 0.78, 95% CI: 0.60-1.02, p = 0.06) showed a trend toward reducing the risk of non-shrinking of EOMs, but did not reach statistical significance. Proptosis (OR = 0.77, 95% CI: 0.60-1.00, p = 0.05) was close to the level of significance, but did not reach statistical significance.

## Discussion

Approximately 20% of GO patients develop restrictive myopathy, characterized by thickening of the EOMs. This thickening may result from swelling of the rectus muscles during the active phase or fibrotic changes in the chronic phase. Restrictive myopathy can manifest as diplopia, significantly impacting an individual’s quality of life and potentially leading to loss of work capacity [7].

Previous studies have shown that retrobulbar injection of GCs can lead to a reduction in EOM volume [4]. This study retrospectively analyzed factors influencing changes in EOMs in GO patients after retrobulbar injection of GCs. Through univariate analysis, LASSO regression analysis, and multivariate logistic regression analysis, we identified several clinical characteristics significantly associated with non-shrinking of EOMs. Ultimately, age and CAS score grouping were found to be important influencing factors for non-shrinking of EOMs. These findings provide clinicians with potential predictors for assessing treatment response in GO patients and aid in optimizing treatment strategies.

The prognosis of GO is influenced by a variety of factors, which can be categorized into endogenous (non-modifiable) and exogenous (modifiable) factors [8]. Numerous studies have shown that patients with elevated TRAb levels and higher CAS values often exhibit higher disease activity, a greater tendency for severe disease progression, poorer prognosis, and a lower likelihood of spontaneous remission [9-12]. In our study, we observed that in GO patients with a CAS score of ≥3, the volume of EOMs was more likely to decrease after retrobulbar injection of GCs. Therefore, for these patients who are more likely to have a positive response to treatment, it may be necessary to consider retrobulbar injection of GCs at an earlier stage. CAS is a scoring system used to assess the degree of disease activity in GO patients [13]. It is considered the most mature and widely used tool for evaluating GO activity, with a total score of ≥3 (out of a maximum of 7 points) indicating an active disease state. In clinical practice, TRAb in the circulation has consistently been found to be associated with GO activity [14,15]. The high expression of thyroid-stimulating hormone receptor (TSHR) in the orbital tissue of active GO patients further supports the key role of TRAb in the pathogenesis of the disease [16-19]. A prospective study independently identified TRAb as a significant risk factor for GO and accurately predicted its severity and prognosis [20]. However, our study did not find an association between TRAb levels and changes in EOM diameter after retrobulbar injection in GO patients.

The clinical manifestations and severity of GO vary significantly across different age groups. For instance, female patients exhibit a bimodal peak incidence at ages 40-44 and 60-64, while male patients show peaks at ages 45-49 and 65-69 [21]. A study targeting Chinese patients (n = 3620) found that the mean age of GO patients was 41.75 ± 13.75 years, and age may be associated with CAS [22,23]. North et al. discovered that age correlates with the CAS score in untreated GO patients, suggesting that age might be an independent predictor of disease activity [24]. Our study revealed that for every 1-year increase in age, the odds of EOMs not shrinking after retrobulbar injection of GCs increased by 11% (OR = 1.11, 95% CI: 1.02-1.20, p = 0.01). This finding indicates that age could be a significant factor influencing the reduction of EOMs. As age advances, the physiological and pathological changes in EOMs may affect their response to GCs treatment.

This study has explored potential predictors of non-shrinking of EOMs through multivariate analysis, suggesting that age and CAS≥3 may be associated with treatment response. These preliminary findings highlight the potential importance of disease activity in influencing outcomes. While proptosis and axial orbit depth showed potential trends, their significance requires further investigation. These results offer a basis for generating hypotheses and guiding future research. They may assist clinicians in considering factors that could influence patient prognosis and treatment planning. Future studies should aim to expand the sample size, explore underlying mechanisms, and assess additional potential predictive factors to refine treatment strategies for GO patients.

## Conclusions

In conclusion, this study has explored potential factors associated with non-shrinking of EOMs after retrobulbar injection of GCs using LASSO and multivariate logistic regression analyses. The results suggest that age and CAS≥3 may be related to the treatment response, indicating the possible influence of age and disease activity. While proptosis and axial orbit depth exhibited certain trends, their statistical significance was not established. These preliminary findings offer a basis for generating hypotheses and warrant further investigation. They may provide clinicians with insights to consider when assessing treatment response in GO patients and developing treatment strategies. Future research should focus on validating these findings with larger cohorts and exploring additional factors to enhance our understanding of treatment outcomes in GO.

The retrospective design and limited sample size of the study may have affected the generalizability and precision of the results. Future research should consider employing a prospective cohort design and including a more diverse patient population to validate the robustness of these findings. Additionally, further exploration of the biological mechanisms underlying these factors may help develop more precise treatment strategies and improve the prognosis for GO patients.
